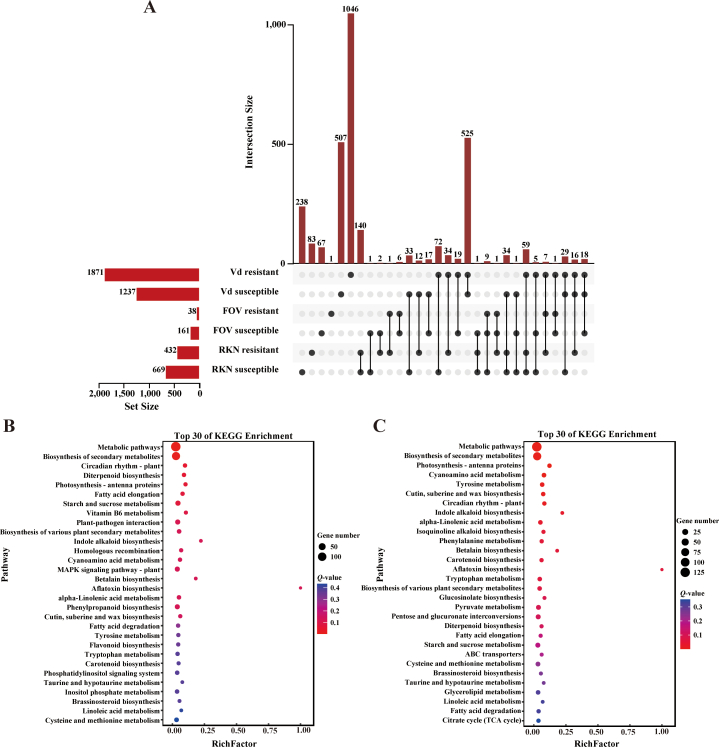# Unraveling genomic regions and candidate genes for multiple disease resistance in upland cotton using meta-QTL analysis

**DOI:** 10.1016/j.heliyon.2023.e18731

**Published:** 2023-07-27

**Authors:** Wen-Qi Huo, Zhi-Qiang Zhang, Zhong-Ying Ren, Jun-Jie Zhao, Cheng-Xiang Song, Xing-Xing Wang, Xiao-Yu Pei, Yan-Gai Liu, Kun-Lun He, Fei Zhang, Xin-Yang Li, Wei Li, Dai-Gang Yang, Xiong-Feng Ma

**Affiliations:** a Zhengzhou Research Base, National Key Laboratory of Cotton Bio-Breeding and Integrated Utilization, School of Agricultural Sciences, Zhengzhou University, Zhengzhou, 450001, China; b National Key Laboratory of Cotton Bio-breeding and Integrated Utilization, Institute of Cotton Research, Chinese Academy of Agricultural Sciences, Anyang, 455000, China; c Western Agricultural Research Center, Chinese Academy of Agricultural Sciences, Changji, 831100, China

**Keywords:** Cotton, Quantitative trait loci, Meta-QTL, Disease resistance, Genomic region

## Abstract

Verticillium wilt (VW), Fusarium wilt (FW) and Root-knot nematode (RKN) are the main diseases affecting cotton production. However, many reported quantitative trait loci (QTLs) for cotton resistance have not been used for agricultural practices because of inconsistencies in the cotton genetic background. The integration of existing cotton genetic resources can facilitate the discovery of important genomic regions and candidate genes involved in disease resistance. Here, an improved and comprehensive meta-QTL analysis was conducted on 487 disease resistant QTLs from 31 studies in the last two decades. A consensus linkage map with genetic overall length of 3006.59 cM containing 8650 markers was constructed. A total of 28 Meta-QTLs (MQTLs) were discovered, among which nine MQTLs were identified as related to resistance to multiple diseases. Candidate genes were predicted based on public transcriptome data and enriched in pathways related to disease resistance. This study used a method based on the integration of Meta-QTL, known genes and transcriptomics to reveal major genomic regions and putative candidate genes for resistance to multiple diseases, providing a new basis for marker-assisted selection of high disease resistance in cotton breeding.

## 1 Introduction

The vast majority of natural fiber in the world are cotton (*Gossypium* spp.) fibers, meaning cotton has a key economic value. Upland cotton (*Gossypium hirsutum* L., 2n = 4x = 52), the most widely cultivated cotton species in the world, has excellent qualities, high yield and is widely adaptable [1]. Extra-long staple (ELS) cotton (*Gossypium. barbadense* L., 2n = 4x = 52), also known as Sea-Island, American Pima, or Egyptian cotton, is instead valued for its superior fiber length, fiber strength and strong resistance to Verticillium wilt (VW) [2].

Plants are subjected to biotic and abiotic stresses caused by plant pathogens, pests, heavy metal, drought, temperature and salt variations. Verticillium wilt caused by the soil borne fungus *Verticillium dahliae* (*V. dahliae*), is a major threat to cotton production [3]. *V. dahliae* can colonize the host body at 48 hours post infection [4], and it causes chlorosis and wilting of the leaves, defoliation, vascular browning and ultimately death of the plant [5]. With the development of molecular biotechnology, researchers have identified several *G. barbadense* L. elite genotypes resistant to Verticillium wilt [6]. Despite these progresses, utilizing the identified genes to improve cotton breeding is still a challenge, as hybrid breakdown occurs in all cases except introgressed breeding lines [7,8].

Fusarium wilt (FW), caused by the soilborne fungus *Fusarium oxysporum* f. sp. *vasinfectum* (FOV), is one of the most destructive cotton diseases. Similar to Verticillium wilt, the pathogen infects cotton roots and colonizes the vascular system, causing the plant to wilt, leaf chlorosis and necrosis, stunting, vascular discoloration, defoliation, and eventually death [9,10]. At present, three dominant resistance genes have been identified: *FOV1* from Pima S-7 (*G. barbadense* L.) [11], *FOV4* from Pima S-6 (*G. barbadense* L.) [12] and *FW*^*R*^ from Zhongmiansuo 35 (*G. hirsutum* L.) [13].

Root-knot nematodes (RKNs) are biotrophic plant-endoparasites of the genus *Meloidogyne* spp*.* that commonly found in tropical and subtropical regions. RKN was firstly discovered in 1885 in cucumber plants. Root-knot nematodes evade plant immunity, disrupt the plant cell cycle, and cause cellular reprogramming and formation of giant cells [14]. RKNs have been shown to make FOV infection easier and hasten the onset of symptoms [15]. When both RKNs and FOV are present in the proximity of the root system, FOV infections significantly increase [16].

Quantitative trait loci (QTLs) have been used to map several key genes that influence cotton agronomic traits [17]. Based on the increased number of identified QTLs, a number of genetic linkage maps of cotton have been constructed. However, most existing genetic linkage maps used to determine disease resistance QTLs do not have high coverage of the cotton genome [18]. The cotton genetic map is estimated to be about 3700–4000 centiMorgan (cM), meaning almost 200 frame markers with average spacing less than 20 cM are needed to provide a full coverage of the genome [19]. The map constructed in a previous study covered 3745.9 cM, estimated to account for 74.92% of the tetraploid cotton genome, but it only had 430 markers [20]. Another study used an interspecific cotton recombinant inbred line (RIL) population derived from 140 interspecific crosses (*G. hirsutum* L. and *G. barbadense* L.) to construct a genetic map 3637 cM long containing 1745 markers [21]. An illumina infinium array (cottonSNP63K) containing 45,104 intraspecific single nucleotide polymorphism (SNP) markers and 17,954 interspecific SNP markers was developed for the first time in *G. hirsutum* L [22,23]. Later, a genetic map 5115.16 cM long containing 2292 markers was constructed [24] and the second SNP array (CottonSNP80K) was created using 100 cotton cultivars [25]. A recent study resequenced 978 different cotton varieties to build a new liquid SNP chip named “ZJU CottonSNP40K” [26], while most previous studies used the “Guazuncho 2 (G. *hirsutum* L.) × VH8-4602 (G. *barbadense* L.)” map [21].

However, research about genetic mapping for breeding is hard due to the difficulty of phenotypic analysis and the lack of genome-wide coverage of gene mapping. In addition, most QTL studies have been carried out on isolated populations and it is not possible to repeat the assessments of disease resistance for the genotypes [27], limiting the reliability of QTL detection [28]. It is therefore necessary to integrate the loci identified in previous researches. Meta-QTL analysis can be effectively performed on QTL data to narrow the QTLs interval. By integrating QTLs from different trials, it is possible to obtain reliable, consistent and stable Meta-QTLs (MQTLs) [29]. Meta-QTL analysis was first used in cotton research to analyze yield, leaf morphology and fiber quality [30]. In rice, three MQTLs associated with rice grain weight were identified and derived from 114 QTLs [31]. Another study in wheat, meta-QTL analysis of QTLs associated with salinity tolerance was undertaken to detect MQTLs using 844 QTLs [32]. In a new study, researchers attempted to map thrips resistance QTLs in cotton for the first time and performed a meta-QTL analysis [33].

The purpose of this study is to conduct a meta-QTL analysis of previously published QTLs for VW, FW and RKN resistance and construct a consensus linkage map based on the genetic map of upland cotton. Our results can be used for cotton molecular breeding and screening of multiple resistance genes in cotton.

## 2 Material and methods

### 2.1 Collection of QTLs data

We carried out a detailed bibliographic investigation on PubMed (http://www.ncbi.nlm.nih.gov/pubmed) and Cotton QTLdb (http://www.cottonqtldb.org) [17] for cotton QTLs related to VW, FW and RKN resistance, published between 2008 and 2022. For each QTL, we retrieved the following data: (i) trait name, (ii) chromosome, (iii) flanking markers or closely linked marker, (iv) peak position and confidence interval, (v) type and size of the mapping population, (vi) likelihood ratio (LOD) score, and (vii) phenotypic variation explained (PVE) or *R*^*2*^ value of QTL. In the absence of a peak position, the mid-point between the two flanking markers was considered as the peak.

If the names of QTLs were unavailable, we assigned names according to the standard nomenclature (“Q” followed by the abbreviated name of the trait, the institute involved, and the chromosome involved). Different QTLs on the same chromosome were distinguished by appending Arabic numerals to the name.

### 2.2 Construction of consensus linkage map

A consensus map was constructed using five available linkage maps involving different types of markers, which have been widely used for QTL mapping: (i) the “Guazuncho 2 (*G. hirsutum* L.) × VH8-4602 (*G. barbadense* L.)” map [21], (ii) the “CCRI36 (*G. hirsutum* L.) × Hai1 (*G. barbadense* L.)” map [34], (iii) the “TM-1 (*G. hirsutum* L.) × Hai-7124 (*G. barbadense* L.)” map [35], (iv) the “TM-1 (*G. hirsutum* L.) × 3–79 (*G. barbadense* L.)” map [36], and (v) the “Yumian-1 (*G. hirsutum* L.) × T586 (*G. hirsutum* L.)” map [37]. Using the BioMercator V4.2 software [38], we run the InfoMap step to check the connection between different maps. We subsequently constructed this consensus linkage map using the Map compilations step.

### 2.3 QTLs projection and meta-QTL analysis

We loaded the map and the QTL files from each study into BioMercator V4.2. Since the map files included the distances between markers on each chromosome, each population of QTLs were mapped to the consensus map separately. Meta-QTL analysis was carried out separately for each chromosome using the Veyrieras two-step algorithm, which is firstly used in cotton. As first step, when the lowest values of the selection criteria were attained in at least three models, the best Meta-QTL model was chosen with the following selection criteria: Akaike information criterion (AIC), AIC corrected (AICc), AIC model 3 (AIC3), Bayesian information criterion (BIC), and Average weight of evidence (AWE). In the second step, MQTLs were generated in accordance with the best model. A MQTL was defined as a genomic area having at least two QTLs. The LOD score and PVE values of MQTL were the means of the LOD and PVE values of the QTLs involved. In order to reduce errors in declaring a QTL, four or more QTLs (false positive rate ≤ 6.25%) in an interval of 25 cM were considered a MQTL region combining [39,40].

Physical locations of individual MQTL were obtained using nucleotide sequences of markers flanking the MQTL. The sequences of markers including simple sequence repeats (SSR), amplified fragment length polymorphism (AFLP) and SNP were retrieved from CottonGen (https://www.cottongen.org) [41]. BLASTN searches were performed on these sequences using the *G. hirsutum* L. reference genome sequences [42].

### 2.4 Verification by previous GWAS studies and reported disease resistance genes

We collected data on disease resistance traits from five GWAS studies published from 2017 to 2021. The data was used to verify the accuracy of predicted MQTL regions. In addition, we collected disease-resistant genes functionally verified in 62 articles from 2005 to 2022. Detailed information about GWAS results and disease resistance genes are listed in Table S2. The physical location of each locus or gene was determined by BLASTN.

### 2.5 Transcriptome analysis of upland cotton response to multiple diseases

We obtained all transcriptome data for disease treatment of upland cotton from the Plant Public RNA-seq Database (PPRD) (http://ipf.sustech.edu.cn/pub/plantrna/) [43]. The R package limma was used to process the data [44]. Differentially expressed genes (DEGs) from transcriptome data sets were filtered using | log_2_ fold change | > 1 and P-adjusted < 0.05. The DEGs of all experiments with different resistance were collected and analyzed. Gene Ontology (GO) term analysis and Kyoto Encyclopedia of Genes and Genomes (KEGG) enrichment analysis of the DEGs in the MQTL intervals were conducted on the GENEDENOVO cloud platform (https://www.omicshare.com/tools/).

## 3 Results

### 3.1 Traits and their associated QTLs

We collected 487 QTLs related to VW, FW and RKN resistance from 31 papers published between 2008 and 2022 (Fig. 1A, Table S1). There were 215 QTLs in the At sub-genome and 272 QTLs in the Dt sub-genome. Chr23 (Dt09) carried the highest number of resistance QTLs (40), followed by Chr14 (Dt02) with 38 QTLs. Chr04 (At04) and Chr25 (Dt06) carried the fewest QTLs, with only five QTLs identified (Fig. 1C). The QTLs included in our meta-QTL analysis mainly focused on VW resistance (68.38%), which is the most severe disease affecting cotton production, followed by FW resistance (24.02%) and RKN resistance (7.60%) (Fig. 1B). Specifically, 333 QTLs were identified for VW, 117 for FW and 37 for RKN.

**Fig. 1:**
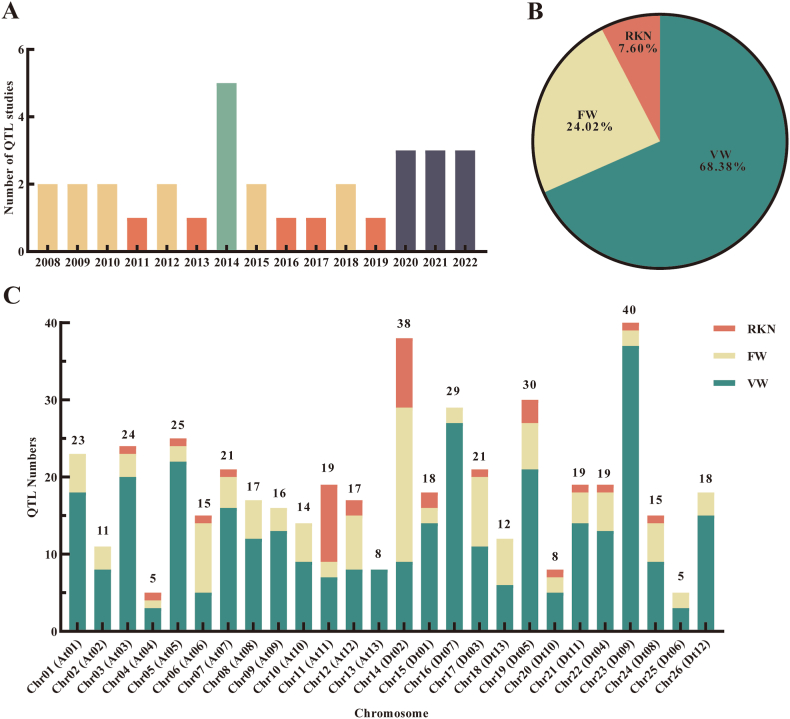
Information of QTLs for biological stress in cotton in previous QTL mapping studies used for meta-QTL analysis. (A) Time distribution of previous QTL mapping studies. (B) Proportion of QTLs for VW, FW and RKN resistance. (C) Distribution of QTLs on chromosomes.

### 3.2 High-density consensus map of cotton

In order to facilitate meta-QTL analysis, we used five maps with strong connectivity to construct a high-density consensus linkage map of cotton. The newly generated consensus map contained 8650 markers, including SSR, AFLP and SNP. The length of the map was 3006.59 cM. The average length of the interval between markers was 0.46 cM. On average each chromosome had 332.69 markers. The chromosome with the highest number of loci was Chr19 (Dt05) (487 markers), while the chromosome with the fewest loci was Chr04 (At04) (179 markers).

**Fig. 2:**
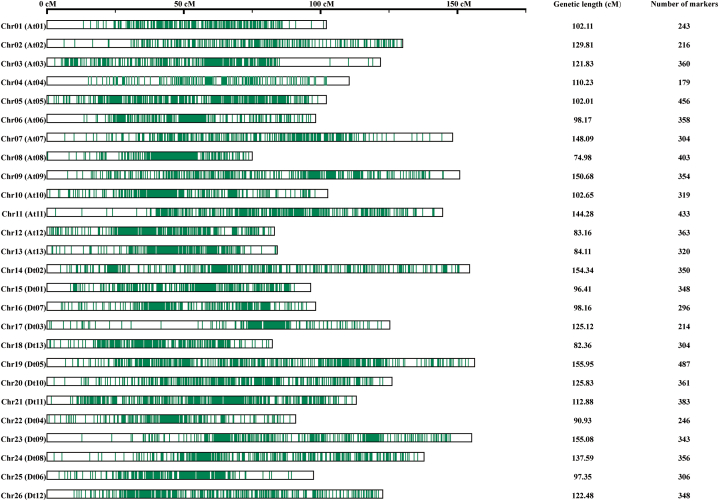
Markers distribution on the consensus genetic map used for meta-QTL analysis.

The longest chromosome was Chr19 (Dt05), measuring 155.95 cM, followed by Chr23 (Dt09), which covered a genetic distance of 155.08 cM. The shortest chromosomes were Chr08 (At06) and Chr18 (Dt13), covering a genetic distance respectively of 74.98 cM and 82.36 cM. The average length of the chromosomes was 115.64 cM. The At and Dt sub-genomes were respectively 1452.11 cM and 1554.48 cM long (Fig. 2).

### 3.3 Identification of Meta-QTL of disease resistance

We used a new meta-QTL analysis strategy to identify MQTLs on the constructed high-density consensus map. Firstly, a total of 487 QTLs were used for meta-QTL analysis, among which 365 QTLs were mapped on the high-density consensus map. The initial position of MQTLs were identified by BioMercator V4.2 (Fig. S1), and the initial MQTLs with multiple QTLs overlaps were merged. Then, 28 MQTLs were identified and derived from 231 initial QTLs. The remaining QTLs were singletons as either they lacked common flanking markers between the initial map and the consensus map, or they did not overlap with any QTL. The MQTLs were named using the standard format, which included the identity of chromosome followed by Arabic numerals for more than one MQTL on the same chromosome (Table 1). The number of MQTLs varied between sub-genomes (13 in At sub-genome and 15 in Dt sub-genome) and among chromosomes.

**Table 1:** Details of 28 MQTLs.

MQTL name	Chromosome	Number of QTLs	Type of hotspot	Peak region (cM)	Start marker	End marker	Start (bp)	End (bp)
MQTL-A01.1	Chr01 (At01)	14	VW	58-85	JESPR0240	NAU3254	34385504	101543362
MQTL-A03.1	Chr03 (At03)	11	VW&FW&RKN	60-85	MUSB1101	MUSS0192	39103387	61925001
MQTL-A05.1	Chr05 (At05)	7	VW	23-47	DPL0641	MUSS0099	2948525	16995738
MQTL-A05.2	Chr05 (At05)	12	VW&RKN	75-90	NAU2296	CM0065	72297115	93042665
MQTL-A06.1	Chr06 (At06)	9	VW&FW	39-60	MUSB0500	MUSB0399	30589238	89059459
MQTL-A07.1	Chr07 (At07)	6	VW	40-65	SWU01338	HAU1763	5833605	17026923
MQTL-A07.2	Chr07 (At07)	4	VW&FW&RKN	70-95	NAU1362	NAU2995	20824707	31884371
MQTL-A08.1	Chr08 (At08)	5	VW&FW	40-65	BNL2961	CGR5521	109517583	123354827
MQTL-A10.1	Chr10 (At10)	5	VW	56-70	UCcg10239_93	MUSB1048	99032791	110135162
MQTL-A11.1	Chr11 (At11)	7	FW&RKN	0-25	MUSS0123	HAU2974	1543478	7039210
MQTL-A11.2	Chr11 (At11)	6	VW&RKN	45-70	DPL0675	JESPR0135	10945699	13752244
MQTL-A12.1	Chr12 (At12)	5	VW&FW&RKN	10-30	CGR5334	CIR0167	2558643	8440833
MQTL-A12.2	Chr12 (At12)	5	VW	50-60	DPL0100	NAU2672	92698217	98983164
MQTL-D02.1	Chr14 (Dt02)	10	VW&FW&RKN	0-25	BNL1403	NAU5499	56797	3751315
MQTL-D01.1	Chr15 (Dt01)	8	VW&RKN	50-75	NAU2985	Gh565	35573287	62528515
MQTL-D07.1	Chr16 (Dt07)	6	VW	35-50	NAU5120	PGML2785	6767431	21516160
MQTL-D07.2	Chr16 (Dt07)	13	VW	70-80	DPL0223	NAU3906	28628170	56375743
MQTL-D03.1	Chr17 (Dt03)	12	VW&FW&RKN	79-90	HAU0195	NAU1167	47133866	49012296
MQTL-D13.1	Chr18 (Dt13)	6	VW&FW&RKN	15-30	HAU1880	Gh443	4298261	10737606
MQTL-D05.1	Chr19 (Dt05)	11	VW&FW&RKN	100-120	Gh447	CM0042	54583246	57436815
MQTL-D10.1	Chr20 (Dt10)	4	VW&RKN	95-110	DC40044	NAU2139	60122658	65277401
MQTL-D11.1	Chr21 (Dt11)	10	VW&FW&RKN	0-25	DPL0320	NAU3374	1632943	5354548
MQTL-D11.2	Chr21 (Dt11)	6	RKN	60-75	PGML1505	DPL0212	21240255	21769977
MQTL-D04.1	Chr22 (Dt04)	4	FW&RKN	0-25	NAU2553	NAU210	481745	3784423
MQTL-D04.2	Chr22 (Dt04)	6	VW&FW	30-50	NAU3824	PGML4137	14658593	56418207
MQTL-D09.1	Chr23 (Dt09)	27	VW&FW&RKN	35-65	NAU1025	TMB0109	2642882	14416367
MQTL-D08.1	Chr24 (Dt08)	6	VW&FW	45-65	MUSS0021	CER0152	4852150	41627448
MQTL-D12.1	Chr26 (Dt12)	6	VW	0-25	DPL0057b	CGR5733	945189	6126291

**Fig. 3:**
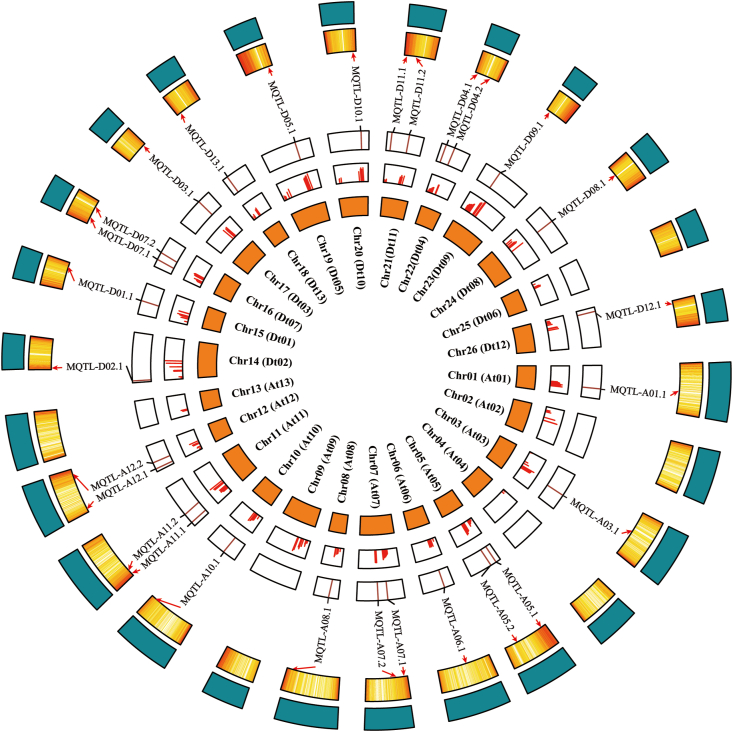
Circular plot showing genome-wide distributions of MQTLs. Circles from the innermost to the outermost represent: genetic map, *R*^*2*^ values of initial QTLs, location of MQTLs on the genetic map, gene density map and the physical map.

The 28 MQTLs were distributed among all cotton chromosomes except Chr02 (At02), Chr04 (At04), Chr09 (At09), Chr13 (At13), and Chr25 (Dt06) (Table 1). Each MQTL contained at least four initial QTLs and the mean interval was 20.61 cM (ranging 10–27 cM). Seven MQTLs (MQTL-A01.1, MQTL-A03.1, MQTL-D02.1, MQTL-D07.2, MQTL-D03.1, MQTL-D05.1 and MQTL-D09.1) contained at least 10 QTLs. A single RKN-associated MQTL was identified on Chr21 (Dt11). Nine MQTLs intervals associated with VW, FW and RKN were found on Chr03 (At03), Chr07 (At07), Chr12 (At12), Chr14 (Dt02), Chr17 (Dt03), Chr18 (Dt13), Chr19 (Dt05), Chr21 (Dt11) and Chr23 (Dt09) (Table 1). Eight VW-associated MQTLs were identified on Chr01 (At01), Chr05 (At05), Chr07 (At07), Chr10 (At10), Chr12 (At12), Chr16 (Dt07) and Chr26 (Dt12). Others were associated with two types of disease resistance. Physical locations of MQTLs were determined based on the reference genome sequence (Fig. 3, Table 1). MQTLs ranged in length from 0.52 Mb (MQTL-D11.2) to 67.15 Mb (MQTL-A01.1), with an average of 15.83 Mb. As expected, the 28 MQTLs contained the higher original *R*^*2*^ (Fig. 3).

### 3.4 Verifying the Meta-QTLs by previous GWAS results and disease resistance genes

**Fig. 4:**
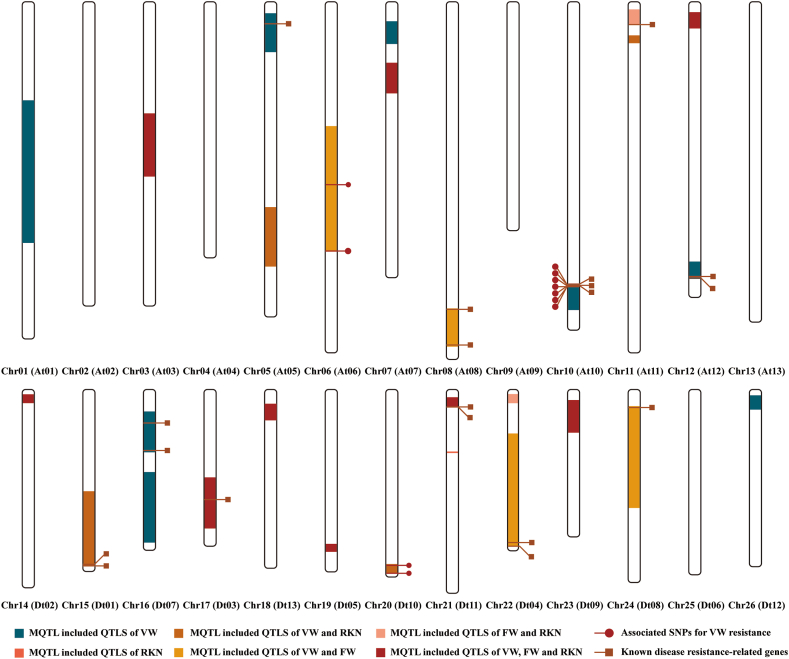
Distribution of MQTLs on cotton chromosomes. Squares are the locations of disease resistance genes, and dots are the locations of SNPs.

In order to determine the reliability of meta-QTL analysis, we used GWAS results and VW, FW and RKN resistance genes published in recent years to verify the MQTLs. As a result, 13 MQTLs overlapped one or more known disease resistance genes or SNPs among the 84 genes (Table S2) and 534 associated SNPs identified through GWAS for VW resistance in cotton (Table S3). Most SNPs were concentrated in the region of Chr21 (Dt11), and there were no MQTLs in this region. Only three MQTLs (MQTL-A06.1, MQTL-A10.1 and MQTL-D10.1) overlapped some SNPs (Fig. 4).

Many crucial genes related to VW, FW and RKN resistance were found in MQTLs regions, including four copies of *GhnsLTPsA10* in MQTL-A05.1, MQTL-A10.1 and MQTL-D01.1 [45], two copies of *CG02* in MQTL-A10.1 [46], *GbSBT1* in MQTL-A03.1 [47], *GhDHS1* in MQTL-A05.2 [48], *GhPAO* in MQTL-A08.1 [49], *GhDSC1* in MQTL-A10.1 [50], *GhPME31* in MQTL-A11.1 [51], *GhMKK10* and *GhSOBIR1b* in MQTL-A12.2 [52,53], *GhPGIP1* in MQTL-D01.1 [54], *GhSOBIR1* in MQTL-D03.1 [52], *GhHb1* and *GhWAKL* in MQTL-D04.2 [55,56], *GhRLP31* in MQTL-D07.1 [52], and *GhPMEI3* and *GhPME2* in MQTL-D11.1 [51] (Table S4). All genes co-localized with MQTLs are related to hormone regulation, reactive oxygen species (ROS), xylem development, pathogen-associated molecular patterns (PAMP), mitogen-activated protein kinase (MAPK) cascades pathway, and synthesis of disease resistant proteins (Table S2).

### 3.5 Predicting candidate genes in multiple disease resistance MQTLs intervals based on transcriptomic data

**Fig. 5:**
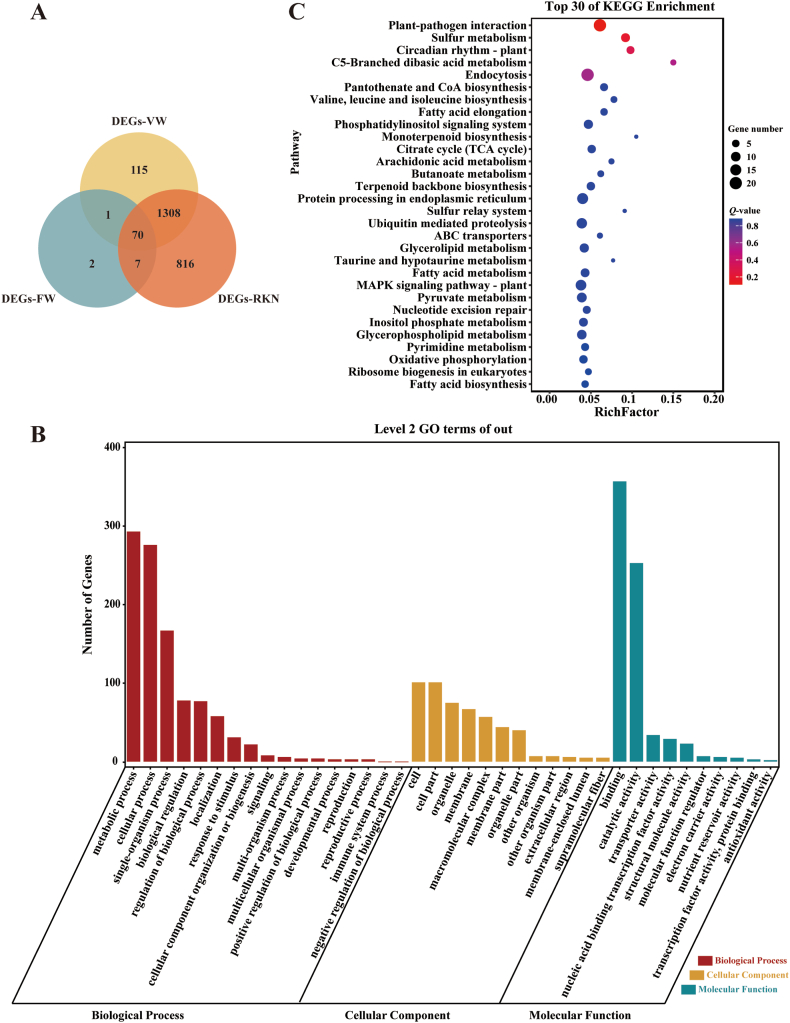
Transcriptomic data associated with multiple disease resistance MQTL regions for cotton resistance to VW FW and RKN. (A) Venn diagram depicting the number of differentially expressed genes (DEGs) involved in three kinds of disease resistance. (B) Level 2 GO terms for DEGs in multiple disease resistance MQTL regions. (C) Top 30 KEGG enrichment pathways for DEGs in MQTL regions.

Interestingly, nine MQTLs (MQTL-A03.1, MQTL-A07.2, MQTL-A12.1, MQTL-D02.1, MQTL-D03.1, MQTL-D13.1, MQTL-D05.1, MQTL-D11.1 and MQTL-D09.1) were found to be associated with VW, FW and RKN resistance. In order to further verify the reliability of the multiple disease-resistant MQTL intervals and analyze the functions of genes contained in the intervals, we re-analyzed all sets of transcriptomic data from public databases relating to treatment of *V. dahliae*, FOV and RKN (Fig. 5).

We firstly performed differential expression analysis of genes in MQTLs associated with resistance to all diseases. We identified 1494 DEGs after *V. dahliae* treatment, 80 DEGs after FOV treatment, and 2201 DEGs after RKN treatment. One DEG was shared between *V. dahliae* treatment and FOV treatment, seven were shared between FOV treatment and RKN treatment, and 1308 were shared between RKN treatment and *V. dahliae* treatment. In addition, 70 DEGs were identified after all treatments (Fig. 5A). We subsequently conducted GO and KEGG enrichment analyses to understand the function of DEGs in MQTLs associated with resistance to all diseases. The most significantly enriched GO terms for molecular function were binding (GO:0005488) (353 DEGs), and catalytic activity (GO:0003824) (250 DEGs). In terms of cell components, the genes were mainly enriched in cells (GO:0005623) and cell components (GO:0044464) (99 DEGs each) (Fig. 5B, Table S5). Among the top 30 pathways identified with KEGG pathways analysis (Fig. 5C, Table S6), the three most enriched pathways were plant-pathogen interaction, sulfur metabolism and circadian rhythm-plant. In addition, MAPK signaling pathway, monoterpenoid biosynthesis and terpenoid backbone, and ubiquitin mediated proteolysis are typical disease resistance pathways in plants.

Furthermore, we performed differential expression analysis of genes in MQTLs associated with resistance to all diseases in resistant and susceptible cotton cultivars. In the resistant cultivars, we identified 1871 DEGs after *V. dahliae* treatment, 38 DEGs after FOV treatment, and 432 DEGs after RKN treatment. In the susceptible cultivars, we identified 1237 DEGs after *V. dahliae* treatment, 161 DEGs after FOV treatment, and 669 DEGs after RKN treatment. Seven DEGs were identified only in all resistant cultivars and one DEGs were identified in all susceptible cultivars (Fig. S2A). We also conducted a KEGG enrichment analysis in resistant and susceptible cotton cultivars. In the resistant cultivars, the DEGs were significantly enriched in plant-pathogen interaction (Fig. S2B, Table S7), but not significant in susceptible cultivars (Fig. S2C, Table S8).

## 4 Discussion

The availability of a genetic map with higher resolution and better genome coverage will represent a valuable resource for QTL mapping and provide useful information for understanding of disease resistance mechanisms in cotton. *G. hirsutum* L. is characterized by high yield and wide adaptability [57,58], while *G. barbadense* L. is highly resistant to diseases [59]. It is therefore representative of many traits to construct a consensus map of *G. barbadense* L. and *G. hirsutum* L. maps. In this study, we built a high-density consensus map long 3006.59 cM which integrates 8650 SNP, AFLP and SSR markers and provides a reliable basis for QTL mapping.

Different mapping populations and molecular markers have been used in recent years to find QTLs with different genetic effects on 26 pairs of cotton chromosomes. In this study, we used 487 QTLs related to VW, FW and RKN resistance for meta-QTL analysis. Recently, meta-analyses have been conducted for a variety of traits in a number of key crops [31,[60], [61], [62], [63]]. Meta-QTL analysis can eliminate the influence of planting environment, population type and genetic background on research results about QTLs, and effectively integrate QTLs data from different backgrounds and environments [64]. Meta-QTL analysis of QTLs has already been carried out about biological stress in cotton [27], and biotic and abiotic stress in cotton [65]. Meta-QTL analysis only by intuitive statistics results in the interval of MQTL not being accurate, while in other plants, newer methods have been developed to locate MQTLs [62]. We used intuitive MQTLs identification methods to identify MQTL, and combined with accurate model calculation methods for further analysis. This meta-analysis method is the first time to be used in the meta-analysis of QTLs associated with disease resistance in cotton. Moreover, we considered more recent QTLs in the meta-QTL analysis than previous studies [27,40,65].

Previous study had found QTLs for VW resistance on almost all cotton chromosomes except Chr10 (At10) and Chr18 (Dt13) [65]. Consistently with previous results, we also identified most QTLs on Chr23 (Dt09) (40) [27]. In addition, we have also identified MQTLs intervals MQTL-A10.1 and MQTL-D13.1, among which MQTL-D13.1 contained QTLs related to both FW and RKN resistance. A meta-QTL analysis on QTLs for VW resistance found 6 QTLs were significantly enriched in the Chr03 (At03) region (93–114 cM) between MUSB0087 and DPL0321, five QTLs in the Chr05 (At05) region (27–47 cM) between DPL0274a and DPL0241, and four QTLs in the Chr07 (At07) region (75–100 cM) between DPL0136 and DC40253 [24]. This result is consistent with the MQTL-A03.1, MQTL-A05.1 and MQTL-A07.2 intervals identified in our study. In addition, MQTL-A05.2, MQTL-D02.1 and MQTL-D11.2 are very similar to the results of another study on significantly enriched MQTLs [27].

Pleiotropic genes are valuable for their potential applications [66]. Since both FOV and VW diseases are caused by fungi, we identified multiple resistance QTLs in cotton for the first time. In this study, we identified nine multiple disease resistance MQTLs, notably Fov4-C19_1_ in the MQTL-D05.1 interval explained 62% of the variation in VRS with a dominance effect of 1.80 [12]. Another region of interest is on chromosome Chr14 (Dt02) (MQTL-D02.1), which was identified as a high-frequency MQTLs region with only involved in RKN disease resistance [27]. A previous study had also identified an important candidate gene for RKN resistance in the qMi-C14 region [67]. GWAS analysis of Fov race 4 resistance have identified other disease-resistance related genes [68]. Candidate resistance genes were found within the QTLs interval, and three genes belonging to the wall-associated kinase (WAK) family were identified on Chr14 (Dt02). This region is also significantly correlated with yield traits [69].

Several well-known genes have been identified accurately in MQTLs. In MQTL-D04.2, *GhWAKL* positively regulates the defense against *V*. *dahliae* by interacting with DnaJ protein to activate salicylic acid (SA) biosynthesis [55]. *GhnsLTPsA10*, co-located with MQTL-A10.1, promotes the phenylpropanoid metabolic flux from the flavonoid biosynthesis pathway to the lignin pathway, and inhibits fungal growth by interacting with fungal membranes [45]. As for MQTL-D11.1, pectin methylesterase-inhibiting protein GhPMEI3 interacts with pectin methylesterases (PMEs) and regulates the expression of a fungal specific polygalacturonase (VdPG1), plays a role in cotton responses to infection by the fungus *V. dahliae* [51]. *GhSOBIR1*, which co-located with MQTL-D03.1, activates the plant resistance pathway by participating in pathogen-associated molecular pattern (PAMP) signaling [52]. These results confirm that the MQTLs region contained key genes involved in response to disease and can be used as candidate regions for map-based cloning of disease resistance genes. In addition, there is a notable region on Chr21 (Dt11) where dense VW related SNPs have been identified in two studies in 2021 [70,71]. Although no MQTL have been identified here, we believe that this region is also of great research value.

GO and KEGG enrichment analyses of DEGs in the MQTL intervals are key to understand the mechanisms of disease resistance. KEGG pathway analysis results were similar to the function of overlapped genes. DEGs in these regions were involved in plant-pathogen interaction, sulfur metabolism [72], MAPK signaling pathway [73], monoterpenoid biosynthesis and terpenoid backbone [74], and ubiquitin mediated proteolysis [75], which are typical disease resistance pathways in plants. In addition, plant metabolic pathways, such as valine, leucine and isoleucine biosynthesis, fatty acid elongation, arachidonic acid metabolism, butanoate metabolism and TCA cycle, might play important roles in the resistance pathways in cotton (Fig. 5C, Table S6).

## 5 Conclusion

The meta-QTL analysis is an approach that has been shown to provide more robust and reliable QTLs. In this study, we constructed a high-density genetic linkage map with a total genetic length of 3006.59 cM, which includes 8650 SSR, AFLP and SNP markers. By summarizing QTL studies associated with VW, FOV and RKN resistance that published in 2008–2022, we identified 28 MQTLs related to biotic stress and mapped them onto the *G. hirsutum* L. reference genome sequences. Nine MQTLs, three in the At and six in the Dt sub-genome, were found to be associated with multiple disease resistance, in which candidate genes were significantly enriched in disease resistance-related pathways. The findings provide a better understanding of the genetic architecture of disease resistance in Upland cotton and will be useful in cotton breeding for high resistance against multiple diseases.

## Author contribution statement

Wen-Qi Huo and Xin-Yang Li: Performed the experiments; Analyzed and interpreted the data; Wrote the paper.

Zhi-Qiang Zhang: Performed the experiments; Analyzed and interpreted the data.

Zhong-Ying Ren, Jun-Jie Zhao and Cheng-Xiang Song: Analyzed and interpreted the data.

Xing-Xing Wang, Fei Zhang, Kun-Lun He, Yan-Gai Liu and Xiao-Yu Pei: Contributed reagents, materials, analysis tools or data.

Wei Li: Conceived and designed the experiments; Analyzed and interpreted the data; Wrote the paper.

Dai-Gang Yang and Xiong-Feng Ma: Conceived and designed the experiments.

## Data availability statement

Data included in article/supp. material/referenced in article.

## Additional information

Supplementary content related to this article has been publish online at [URL].

## Declaration of competing interest

The authors declare that they have no known competing financial interests or personal relationships that could have appeared to influence the work reported in this paper.

## Figures and Tables

**figs1:**
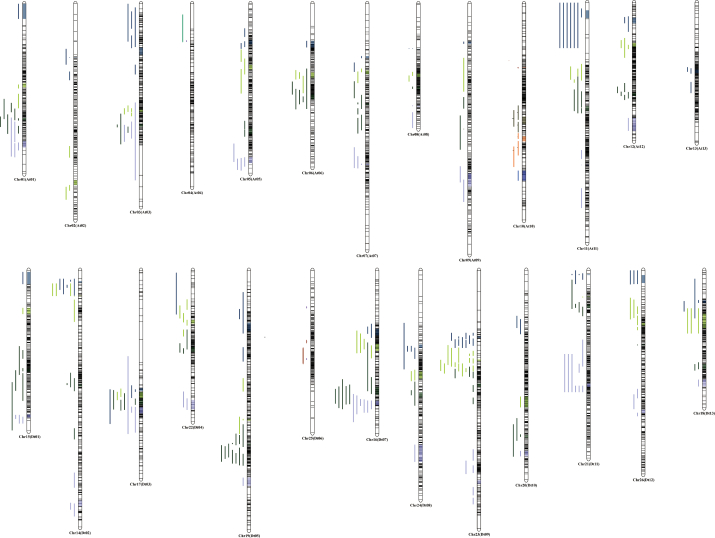


**figs2:**